# Supplementary material for: Are carbon emissions trading and green financial instruments synergistic? -Comprehensive quantitative research based on content analysis
Source: PLoS One. 2024 Mar 7;19(3):e0298601. doi: 10.1371/journal.pone.0298601 (PMC10919601; doi:10.1371/journal.pone.0298601)
Supplement: S1 Data — (ZIP) [file pone.0298601.s001.zip › Data/地级市Z_F工作报告.zip!/║╙▒▒/▒ú╢¿╩╨/└┤╫╘╣1⁄2╓┌║┼í╛┬φ┐╦╩2╛▌═°í┐_2017.docx]

各位代表：

现在，我代表市人民政府，向大会作工作报告，请予审议，并请市政协委员和列席会议的同志提出意见。

总结本届政府工作

保定跨越崛起和复兴其时已至其势已成其兴可待

十四届人大一次会议召开以来的四年，全市上下在省委、省政府和市委的坚强领导下，坚持新理念，抢抓新机遇，应对新考验，引领新常态，培育新动能，不忘初心、砥砺前行、奋力拼搏，圆满完成了既定目标任务，为全面建成小康社会奠定了坚实基础，谱写了朝着保定跨越、崛起和复兴目标迈进的历史新篇章。

四年来，我们心无旁骛抓发展，千方百计稳增长，扩增量、保总量、提质量，综合实力跨上新台阶。全市生产总值突破三千亿元大关（不含定州，下同），由2481.3亿元（2012年）增加到3110.4亿元（2016年），年均增长7.5%；人均生产总值由2.45万元增加到2.99万元，年均增长6.9%；一般公共预算收入由146.6亿元增加到222亿元，年均增长10.9%。新增财政收入10亿元以上的县（市、区）7个、突破5亿元的10个。粮食生产实现“十二连丰”，连续八年突破100亿斤。规模以上工业增加值达到1010.8亿元，年均增长7.3%；固定资产投资完成2620.7亿元，年均增长11.2%；社会消费品零售总额1669.3亿元，年均增长11.7%。综合实力和发展竞争力不断增强，重返全省第一方阵。

四年来，我们凝神聚力抓提升，坚定不移调结构，强支柱、补短板、促转型，产业升级迈出新步伐。全市三次产业结构由12.6∶55.4∶32调整为11.8∶49.6∶38.6。农业产业化经营率达到67.8%，建成现代农业园区100万亩，省级农业园区数量、规模均居全省第一。五大主导产业增加值完成620.2亿元，年均增长9.6%。大王店六村征迁联建攻坚行动顺利完成，工业园区实现量产，长城汽车年销售突破百万辆、销售收入千亿元、实现利税各百亿元。曲阳、涞源等县24个山地光伏发电项目让太行山变成了太阳山。高新技术产业增加值完成451.8亿元，年均增长13.4%。去年旅游总收入达到757亿元，快递业务量超过1.8亿件。银行、保险机构分别达到35家和54家，上市企业81家、直接融资651亿元、实现县市区的全覆盖。服务业增加值达到1199.4亿元，年均增长9.4%，经济贡献率达到48.5%，成为经济增长的第一支撑力。

四年来，我们积极主动抓机遇，实施协同发展战略，强基础、做平台、深对接，京津保率先联动发展实现新突破。继深圳经济特区和上海浦东新区之后又一具有全国意义的新区——河北雄安新区落地保定，非首都功能集中承载地规划建设全面启动。交通一体化进程加快，京石高铁建成通车，津保铁路百年梦圆，京昆、张石、保阜高速全线贯通，京港澳高速改扩建全面完成，高速公路通车里程962公里，京津保地区1小时交通圈基本形成。一批重大产业承接平台加快建设，京津保产业合作项目达到516项，竣工93项，在建118项。保定·中关村创新中心入驻企业86家，清华启迪之星（保定）入驻36家。白沟大红门服装城入驻商户1600家，蠡县裘皮小镇入驻企业商户1300余家，高碑店新发地物流园日交易额过亿元，北京儿童医院托管市儿童医院，301医院涿州基地即将运营，北京八一学校保定分校建成使用，教育合作项目达到108个，59家医院与京津79家医疗机构开展合作。涞水中电科产业园、安国中药都、清苑中汽零基地、唐县北大未名通天河产业园等一批重大园区建设加快。中国动力、涿州中关村科技产业园等正式签约。与兵装、中航、中船重工等36家央企合作的78个项目落地。探索形成的园区共建、筑巢引凤、政企牵手、企业联姻、托管合作、创新联盟六种区域合作模式初见成效。

四年来，我们大胆探索走新路，实施创新驱动战略，出政策、聚要素、增动能，跨越赶超点燃新引擎。与科技部、中科协、中科院等深度合作，建成产业创新联盟26家、院士工作站21家、学会服务站56家、科技创新平台273个。全市科技型中小企业达到6263家，高新技术企业达到241家，科技贡献率达到52%，创新企业数量和科研投入位居全省前列，科技管理工作连续两年全省第一。建成众创空间58家，在孵创业团队2120个，创新创业蔚然成风。总投资50亿元的长城汽车新技术中心投入使用，中关村“一中心一基地一园区”[1]、白洋淀科技城和保定深圳科创基地建设加快推进。保定经开区获批省级创新型特色园区示范试点，涿州、白洋淀国家农业科技园区获科技部批准。我市创新驱动的做法受到国务院办公厅通报表扬。

四年来，我们坚持绿色发展不动摇，常抓不懈治污染，用实招、出重拳、强攻坚，生态环境治理取得新进展。持续开展“十大攻坚行动”[2]，煤改气、煤改电、煤改清洁能源，主城区三环以内基本实现“散煤清零”，京津冀禁煤区九县（市）完成改造401个村21万户。全市完成燃煤锅炉替代改造8394台。主城区90%以上餐饮单位安装了油烟净化装置，骨干道路实现机械化湿式清扫，建筑工地全面落实降尘措施。在全省率先制定并实施了山水林田湖生态整体修复规划。投资5.6亿元加强白洋淀水域综合治理，淀区水质持续改善。完成人工造林、封山育林260万亩，全市森林覆盖率达到28.2%，市区绿化覆盖率达到42%，分别提高5.6个和5个百分点。2016年空气质量综合排名退出全国重点监控城市倒数第一，PM2.5浓度均值比2013年下降31%，空气质量达标天数155天、比2013年增加了81天。

四年来，我们坚持统筹协调发展，大力推进新型城镇化，扩城区、强城镇、带农村，城乡面貌发生新变化。实现行政区划调整，中心城区面积扩大到2531平方公里，常住人口增加到285.8万人，迈出了破解小马拉大车历史难题的关键一步。朝阳大街南延、北三环东延、白洋淀大道建成通车，乐凯大街南延、复兴路西延开工建设，高铁片区、东湖片区、西湖体育新城建设加快推进。实施城市管理“6S”行动[3]，城市环境明显改善。总投资898亿元的426项县城建设“七个一”工程[4]成效明显，省级园林县城创建基本实现全覆盖。白沟镇入选国家新型城镇化综合试点。完成乡改镇、镇改办事处36个，全市城镇化率达到49%，提高7.5个百分点。建成省级美丽乡村1847个，白洋淀、涞涞易示范片区旧貌换新颜。白石山成功创建5A景区，野三坡中国经济论坛大家云集，成功承办河北省首届旅发大会，“京西百渡”成为保定新名片。全面实施南水北调配套工程，成为全省首个用上长江水的设区市。公路通车里程达2.2万公里、居全省第一。

四年来，我们坚持不断解放思想，深化改革扩大开放，破障碍、降成本、优环境，经济社会发展释放新活力。全面改革不断深化，承接省委、省政府部署的93项改革任务全部完成，27个改革试点稳步推进，市本级108项改革任务取得阶段性成效。扎实推进供给侧结构性改革，“三去一降一补”成效显现，2016年商品房待售面积（133.1万平方米）比上年下降50.3%。“营改增”同比减税22.7亿元，2.5万纳税户受益。国资国企改革不断深化，市属经营性资产统一监管，大世界商业城、蠡县信用社等遗留多年的问题成功化解。积极推进商事制度改革，企业注册登记更加便捷，各类市场主体由24万户增加到53万户。农业农村、教育卫生、财税体制等重点领域和关键环节改革取得积极进展。白沟箱包市场被列为国家市场采购贸易方式[5]试点。积极面向国内外招商引资，举办了百家央企进河北、首届世界冀商大会、白洋淀国际服装文化节、第四届中国市场大会（白沟）和赴北京、天津、深圳推介招商等一系列大型活动，同仁堂、天士力、百威啤酒等一批重点项目落户保定。冀欧国际货运专列开通运营。全市累计实际利用外资23.1亿美元。

四年来，我们坚持共享发展理念，不遗余力改善民生，抓脱贫、保就业、增福祉，社会事业稳步发展，人民生活水平得到新提高。财政投入向民生倾斜，累计支出1561.6亿元，占一般公共预算支出的80.8%。实施五大精准脱贫工程[6]，创新十大扶贫模式[7]，全市贫困人口减少106.5万人，贫困发生率下降12.1个百分点。城乡居民人均可支配收入分别达到25680元和11612元，增长34.8%和50.9%。城镇新增就业人口37.4万，开工建设保障性住房12.16万套，改造农村危房7.3万户，273.2万农村人口饮水安全问题得到解决。医疗和养老保险实现全覆盖，城乡居民基本医疗保险财政补助由每年240元提高到420元，城乡居民基础养老金最低标准由每月55元提高到80元，市县“多院合一”的民政服务中心全部建成，被确定为河北省唯一的全国和谐社区建设示范城市。义务教育投资56.8亿元，新改扩建学校123所，山区义务教育寄宿制学校建设三年行动计划全面完成。安全生产、食品药品安全形势稳定向好，法治保定、平安保定建设扎实推进。“保定舰”正式加入海军战斗序列，全国双拥模范城实现“八连冠”，军民融合发展走在全省前列。精神文明建设成果丰硕，“文化润古城”系列活动丰富了群众生活，保定学院西部支教群体、“太行新愚公”李保国、“油条哥”刘洪安、三八红旗手标兵王焕荣成为全国模范典型。

四年来，我们坚持为民务实清廉，努力加强自身建设，转职能、提效率、强服务，依法行政能力得到新增强。推进“放管服”改革，市本级共取消行政审批事项605项，下放73项，精简比例为57%。出台“四份清单一个标准”[8]、完善“两个代办”升级版、构建市县乡村四级服务平台的做法在全省推广。市县两级政府全部聘任法律顾问，市政府成立参事室，政府决策更加科学民主。首次行使地方立法权，出台了“两条例一办法”[9]。狠抓反腐倡廉，落实“一岗双责”，把行政权力关进制度的笼子，机关作风明显好转。自觉接受人大法律监督和政协民主监督，共办理人大代表建议354件、政协提案1562件，办复率100%。主动接受社会监督和舆论监督，累计受理群众来信来电、短信和网络留言51万余件（条），办结率90%以上。

各位代表！四年在历史的长河里只是短暂的一瞬间，但是过去的四年在保定发展的史册中留下了浓墨重彩的一笔。这四年，我们沐浴在总书记的亲切关怀下，置身于协同发展的重大机遇中，“京津保”写入《规划纲要》，河北雄安新区这一千年大计、国家大事、重大历史性工程花落保定，开启了保定发展的新纪元，为实现保定复兴插上了腾飞的翅膀。这四年，我们从区划调整入手，统筹城乡、提质增色，城市颜值全面提升，美丽乡村绽放异彩，期待已久的大保定正在蓄势崛起。这四年，我们主动融入国家战略，聚力推进协同创新，打造创新创业新载体，培育经济发展新动能，全国创新驱动发展示范市建设风生水起。这四年，我们从百姓呼声最迫切的事情做起，铁腕治霾，精准扶贫，实事惠民，打响了一场改变生产生活方式的攻坚战，得到广大人民群众的充分理解和支持，让我们感恩感动，充满信心和力量。这四年，我们励精图治，“实”字为先、“干”字当头，全市上下政通人和，干部群众激情创业，首届省旅发大会精彩、震撼、圆满，锤炼出“旅发精神”，创造了“保定速度”，重塑了保定形象和尊严，成为我们做好今后工作的强大动能。四年来不平凡的发展历程，充分印证了市第十一次党代会的正确判断：保定的跨越、崛起和复兴，其时已至，其势已成，其兴可待！

各位代表！过去四年成绩的取得，是市委总揽全局、正确领导的结果，是市人大、市政协有效监督和大力支持的结果，是广大干部群众团结协作、共同奋斗的结果。在此，我代表市政府，向全市人民，向人大代表、政协委员，向各民主党派、工商联、各人民团体和社会各界人士，向老干部，向驻保单位、解放军指战员、武警官兵和政法干警，向所有关心支持保定发展的朋友们，致以崇高的敬意和衷心的感谢！

各位代表！我们也深知，我市经济社会发展中仍存在不少的问题，政府工作还有很多不足。一是思想不够解放，面对新形势新问题，政策不活、办法不多、措施不硬，引领把握新常态的能力有待进一步加强；二是总体实力不强，实体经济缺少四梁八柱，县域经济发展不平衡，城乡居民收入偏低，贫困人口多的“短板”比较突出，脱贫任务还很重；三是城镇化水平较低，城市功能不够完善、管理不够精细，部分道路交通拥堵，马路市场还需大力整治；四是重污染天气时有发生，大气污染防治形势依然严峻，生态恢复与环境保护任务十分艰巨；五是公共服务能力不足，在住房、教育、医疗、养老等方面还有不少让人民群众不满意的地方；六是营商环境需要进一步优化，少数干部懒政怠政、推诿扯皮，不作为、慢作为，一些领域腐败问题仍有发生。对此，我们一定坚持问题导向，把群众的关心化为我们的信心，把百姓的期盼作为努力的方向，不回避不护短，真用心真作为，切实解决问题，不负人民重托。

今后五年的奋斗目标

加快建设创新驱动经济强市全面建成小康社会

全面建成小康社会，是中华民族伟大复兴中国梦的第一个百年奋斗目标。加快建设创新驱动经济强市，是省第九次党代会为我市确定的发展定位。刚刚闭幕的市第十一次党代会，制定了建设创新驱动经济强市、推动京津保地区率先联动发展、全面建成小康社会、加快实现保定复兴的宏伟目标、发展路径和战略举措，发出了加快实现保定跨越崛起和复兴的动员令。站在新的历史起跑线上，伴随着河北雄安新区建设的铿锵步伐，实现全市一千多万人民的新梦想、新希望，我们必须深入贯彻习近平总书记系列重要讲话精神和治国理政新理念新思想新战略，在跨越发展中崇尚创新、注重协调、倡导绿色、厚植开放、推进共享；必须全面落实习近平总书记在雄安新区规划建设座谈会上的重要讲话精神，落实“四个加快”“六个扎实”总体要求和“三个扎扎实实”的工作要求[10]，在对接京津、服务京津中加快发展自己，在改革创新、开放合作中加快实现新旧动能转换，在治理污染、修复生态中加快营造良好人居环境，在脱贫攻坚、推进共享中加快提高人民生活水平；必须坚定走加快转型、绿色发展、跨越提升新路，推进更高质量、更高效率、更为公平、更可持续的发展，实现发展阶段、发展层次整体跨越和提升；必须进一步做好服务、承接、支撑三件大事，服务京津和雄安新区建设，打造非首都功能疏解的支撑点、京津产业转移的承接地、外资进入京津乃至北方市场的桥头堡；必须坚持用好五个关键词，唱响实干实政、决战决胜主旋律，扎实务实抓落实，实干苦干求发展，重塑保定尊严、再创大市辉煌。

今后五年政府工作的指导思想是：全面贯彻党的十八大及中央、省和市委系列会议精神，坚持以人民为中心的发展思想，主动认识、把握和引领经济发展新常态，大力推进供给侧结构性改革，服从服务支持保障河北雄安新区建设，围绕市第十一次党代会确定的目标任务，深入实施“协同发展、创新驱动、环境支撑”三大主体战略，全力打好城市做大、产业做强、生态做美、民生做富四场硬仗，为建设创新驱动经济强市，推动京津保地区率先联动发展，全面建成小康社会、加快实现保定复兴而奋斗。

今后五年，我们要和全国人民一道，完成“十三五”目标，全面建成小康社会，让全市人民过上更加殷实富裕的小康生活。今后五年，我们的核心目标是：加快建设创新驱动经济强市。创新驱动关键在一个“新”字，要新在高端理念上、新在研发能力上、新在动能培育上、新在体制机制上、新在环境支撑上；经济强市关键在一个“强”字，要强在核心竞争力上、强在产业支撑力上、强在新兴成长力上、强在政策保障力上、强在综合实力上。我们的中心任务是：全力支持雄安新区建设，推动京津保地区率先联动发展。“率先”，就是在对接融入上率先，在承接落地上率先，在增比进位突破上率先；“联动”，就是在推进交通一体化上联动，在打造生态宜居、高品位城市上联动，在提升公共服务品质上联动；“发展”，就是全面建成小康社会、加快实现保定复兴。今后五年，我们要经过坚持不懈的努力，推动经济社会发展实现六个显著提升。

（一）综合经济实力显著提升。坚持走工业强市、服务业兴市之路，实体经济支撑作用明显增强。促进一二三产融合发展，新型工业化、信息化、城镇化、农业现代化的步伐加快。实施“双千双百”工程[11]，城市经济、县域经济、园区经济实力显著增强。生产总值年均增长9%、达到5000亿元左右，一般公共预算收入年均增长10%、达到350亿元左右，服务业增加值占GDP比重接近50%，农业产业化经营率达到70%以上，主要经济指标增速明显高于全国、全省平均水平，经济增长保持中高速，发展水平迈向中高端，综合实力稳居全省第一方阵。

（二）协同创新活力显著提升。以“三争一创”统领协同发展，争当国家级创新型城市，争当全国创新驱动发展示范市，争当京津冀协同创新共同体的排头兵，与河北雄安新区牵手，开创京津保地区率先联动发展的新局面。石保廊全面创新改革试验工作和京南国家科技成果转移转化示范区建设取得重大进展，建成全国重要的产业创新基地、科技成果转化基地和战略性新兴产业聚集区。高新技术产业增加值占规上工业增加值比重达到51%以上，科技进步贡献率达到60%以上。

（三）改革开放成效显著提升。持续推进供给侧结构性改革，市场配置资源的决定性作用得到充分发挥，发展动能不断增强。持续推进行政体制、商事制度、财税金融等改革，各类市场主体年均增长10%，市场主体活力明显增强。创新招商引资机制，深度融入“一带一路”，借势雄安新区建设，推进高水平双向开放，利用外资年均增长9%，经济外向度不断提高。营商环境对标京津、消除落差，打造政策洼地、开放高地，让更多人才走进保定创业，让更多企业家投资保定发展。

（四）城乡发展水平显著提升。推进新型城镇化和城乡统筹示范区建设，加强与河北雄安新区规划衔接，打造京津冀区域性中心城市和全国性综合交通枢纽城市。彰显古城魅力，建设现代新城，中心城市格局进一步优化，立体交通体系初步成型，主城区与新三区融合发展，与雄安新区互动发展，辐射带动能力显著增强。县城建设扩容提质，城市功能日趋完善，建成一批微中心和卫星城，建成10个国家级园林县城、60个特色小镇[12]。加快乡改镇、村改居，每年新增城镇人口33万人以上，常住人口城镇化率达到63%以上，形成“双城带动”[13]、县城联动、特色小镇互动、城乡统筹的新格局，让保定在世界级城市群建设中迅速崛起。

（五）生态环境质量显著提升。秉承生态就是民生、生态就是财富的理念，持续开展“蓝天”“碧水”“净土”“绿满太行”四大行动。扭住“压煤、抑尘、控车、减排、迁企、增绿”六大主要环节，打好蓝天保卫战，PM2.5平均浓度明显下降，大气污染稳定退出全国后10名。环境治理和生态修复取得重大突破，全市森林覆盖率达到36%，市区绿化覆盖率达到45%，水功能区水质达标率达到80%，让保定青山常在、绿水常流、空气常新，环境魅力不断增强。

（六）公共服务能力显著提升。坚持以人民为中心，推进公共服务均等化，改善人民生活，增进人民福祉。贫困县、贫困村、贫困人口全部脱贫出列。居民人均可支配收入年均增长11%以上，城镇登记失业率控制在4.5%以内，城乡低保标准高于全省平均水平，社会保障全面覆盖，住房保障体系基本形成。科教文卫事业繁荣发展，养老服务体系不断健全。党风政风社会风气持续好转，公民思想道德和社会文明程度明显提升。建成全国食品安全城市，全面打造健康保定、平安保定、法治保定、诚信保定。

各位代表！器大者声必宏，志高者意必远。宏伟的目标召唤着我们，新征程的号角鼓舞着我们。我们一定坚定信心，勇往直前，矢志奋斗，努力把宏伟蓝图早日变成美好现实！

2017年的主要任务

聚焦聚力精心精细做好八个方面重点工作

今年是实施“十三五”规划的重要一年，是供给侧结构性改革的深化之年，也是实现京津冀协同发展近期目标最为较劲的一年，做好今年的政府工作至关重要。全市经济社会发展的主要目标是：生产总值增长8%以上，规模以上工业增加值增长7%，服务业增长10%，一般公共预算收入可比增长10%，固定资产投资增长12%，社会消费品零售总额增长10%左右，出口总值增长7%，实际利用外资7.2亿美元。万元生产总值能耗、PM2.5、化学需氧量、二氧化硫、氨氮、氮氧化物减排完成省定目标。城乡居民人均可支配收入分别增长8.5%和10.5%。

（一）强力推进协同发展，强化对接服务。全力唱好京津保率先联动发展“重头戏”，确保实现协同发展近期目标，在对接京津、服务新区中加快发展自己。

积极服务河北雄安新区建设。设立雄安新区，是以习近平同志为核心的党中央深入推进京津冀协同发展作出的重大决策部署，是重大的历史性战略选择，是千年大计、国家大事，对于集中疏解北京非首都功能，探索人口经济密集地区优化开发新模式，调整优化京津冀城市布局和空间结构，培育创新驱动发展新引擎，具有重大现实意义和深远历史意义。设立雄安新区，是党中央对河北人民、对保定人民的关怀和支持，也是我们面临的千载难逢的重大机遇。我们一定切实增强“四个意识”，做到“四个坚决”（服从、支持、借势、保障），自觉服从大局，全力支持配合，精心服务保障，主动对接融入，全面接受辐射，不断迈出京津保率先联动发展的新步伐。

推进三个重点领域率先突破。打造区域重要交通枢纽，加强与京津和雄安新区交通互联互通，共建快捷高效交通网，年内荣乌、曲港高速全线通车，加快太行山高速、石津高速（保定段）建设。加快建设廊涿、固保城际铁路，推进京石城际、京太（保五忻）、保张、保沧铁路前期，构建“六射两连”[14]铁路网。谋划通用机场建设。促进生态共建共享，落实京津冀生态保护补偿和大气污染联防联控措施，抓好京津保平原生态过渡带建设，依托河湖水系和交通干线，大规模植树造林，开展生态修复绿化，建设绿色生态隔离带。创新产业合作模式，发展高端高新产业，积极承接产业转移，促进我市主导产业加快转型升级。

深化与京津的项目合作。把项目作为承接非首都功能和产业转移的关键来抓。以京津为主攻方向，引资引智并重，深化与国家部委、央企总部、高等院校、科研院所的对接合作，抓好项目筹划、筛选、储备，充实完善对接京津项目库。对已签约的200多个合作意向，专人盯办，压茬推进，早日落地。突出抓好36个省重点和28个重大产业项目，实行“四个一”[15]管理，实现快开工、快建设、快竣工、快达产。推进涿州中船重工海洋装备科技产业园、莲池深安·智谷、启迪环保科技城、东湖金融港、高新区国鹰无人机生产基地、涞水北京公交智造、博野三棵树生产基地、满城生物制药产业园、易县航天民品产业园及抽水蓄能电站等重大项目取得明显进展。

（二）强力推进协同创新，增强发展新动能。深入实施创新驱动发展战略，实施创新型保定建设15项工程[16]，加快培育保定复兴的核心动能。

打造协同创新共同体。积极推进石保廊全面创新改革试验区、京南科技成果转移转化示范区、全国产业转型升级试验区政策落地。强化科技创新应用和示范推广能力，共建科技园区、创新基地、技术市场、转化基金和创新联盟。深化与京津高校院所的科技合作，形成共建共享、协作配套、统筹互助机制，最大限度地吸引京津技术到我市孵化转化。借势雄安新区集聚效应，积极吸纳创新要素资源，打造创新创业集群。

搭建协同创新大平台。加快白洋淀科技城、保定深圳科创产业基地、中关村“一中心一基地一园区”和丰台园满城分园、石景山园定兴分园、中国动力产业园建设，打造一批科技成果孵化转化基地，全年新增各类创新平台20家以上。发挥高新区创新政策引领带动作用，推动一区多园建设。抓好保定经开区、徐水经开区、安国中药都、唐县北大未名、涿州中关村、京涞新城、望都光翌等重大产业园区。支持各县与京津共建产业园区，提高综合承载能力和协作配套能力。

培育协同创新主力军。加快科技型企业培育，促进新兴业态成长。培育创新型领军企业，加快建设长城新技术中心、高碑店国家建筑节能国际创新园、涞水中电科产业园、风帆研究院、莲池邦泰高分子新材料应用技术研究中心，提升市场主体的创新能力。引进培育一批高新技术企业，年内新增35家以上。抓好苗圃、雏鹰、科技小巨人和企业上市四大工程，推动科技型中小企业持续增长，新增1000家以上。扶持一批创客群体，鼓励社会力量参与众创空间的建设和运营管理，吸引京津众创空间来保设立分支机构。加强校地校企合作，支持驻保高校发展，用好驻保高校创新资源。引进培养一批领军人才、企业家人才、高技能人才和创新团队。探索面向人才群体的住房保障制度，让更多人才留在保定成就梦想。

（三）强力推进实体经济发展，夯实强市基础。积极落实省产业转型升级十大专项行动[17]，促进资源要素向实体经济聚集。

做强工业支撑。以创建“中国制造2025”示范试点城市为抓手，以智能制造为主攻方向，加快构建“保定制造业253”体系。做强做优支柱产业，完善汽车产业链条，打造汽车产业集群，支持长城汽车打造世界级领军企业，依托大王店国际汽配园、中汽零产业园和中兴、立中、风帆、凌云等企业，建设汽车零部件生产基地和出口走廊；完善“电字号”产业链条，打造“中国电谷”升级版，支持英利、国电、天威、四方三伊、荣毅等龙头企业发展，构建变电储电、智能电网、光电网、智慧能源＋新材料、电子信息、节能环保等产业集群。支持乐凯、奥润顺达、巨力、晨阳等优势骨干企业放量增长，打造一批行业领军企业。落实“个转企、小升规、规改股、股上市”政策，年内每县新增规上工业企业5—10家，全市新增150家以上。培育壮大新兴产业，加快发展以数控机床、智能机器人、精密传感器为重点的高端装备制造，以氮化镓、碳化硅、石墨烯、硼材料等为重点的新材料，以大数据、云计算、集成电路等为重点的新一代信息技术，以现代中药、新型制剂、医疗设备等为重点的生命健康产业，以中电科空间信息、航空航天、海洋装备等为重点的军民融合产业。改造提升传统产业，推动高阳纺织、蠡县皮毛、曲阳石雕、白沟箱包、望都辣椒、满城纸品、清苑燃香、顺平肠衣等特色经济上档升级。发挥传统优势，促进建筑业持续健康发展。

做强现代农业。深化农业供给侧结构性改革，稳定粮食、提升蔬菜、增加果药、扩大饲草料，保障供给质量，增加农民收入，提高农业农村综合效益和竞争力。紧盯京津市场，加快现代农业园区建设，年内新增精品特色园区10个、县级园区30个、市级园区25个，省级以上园区达到20个。抓好涿州国家农业科技园区建设，积极争创保定（涿州）国家农业高新技术产业开发区，推进顺平等西部山区果品产业带和环首都现代农业科技示范带建设。推广“互联网＋品牌”应用模式，实施“一县一品一牌”培育计划，加大“三品一标”[18]认证和支持力度。深入实施农产品加工倍增计划，支持农民合作社、家庭农场等新型经营主体的发展，重点支持100家种养加龙头企业，新增农村新型经营主体1500家以上。加快土地流转，引导农民通过村组互换并地等方式，实现按户连片耕种。加快发展农村电商、休闲农业、乡村旅游等新型业态。

做强现代服务业。坚持生产性服务业与生活性服务业并重，壮大规模，提档升级。加快发展旅游业，完善“京西百渡”后续项目建设，办好高碑店、定兴、白沟新城首届市级旅发大会，打造京南小镇特色旅游体验区，提前做好第二届（顺平、满城、竞秀）和第三届（曲阳、阜平、唐县）市旅发大会谋划、筹备工作。积极推进易县清西陵、狼牙山、阜平天生桥、满城汉墓5A级景区创建。发展全域旅游，构建“京津保”旅游大格局。做强金融支撑，推进保定银行廊坊分行尽早挂牌开业。加强政银保企对接，鼓励创新金融产品和保险服务，引导扩大信贷投放。培育上市后备企业，支持企业直接融资，每县新增挂牌上市企业1—2家。加强市县两级投融资平台建设，每县融资2亿元以上，全市融资超过100亿元。推进金融扶贫示范区建设，支持阜平申报国家普惠金融试点，推进新型农村合作金融试点、民间资本管理公司试点，择机做好政府发债。加快社会信用体系建设，加强金融监管，防控金融风险。大力发展现代物流业，加快与互联网、物联网融合，打造电子商务平台和第三方物流企业，建设大宗商品交易中心和大型农产品标准化市场。支持农产品电商平台和乡村电商服务站点建设。完善“快递下乡”工程，加快快递园区建设，打造国家二级快递枢纽。抓好白沟国家级市场采购贸易方式试点工作。推动体育休闲、文化创意、影视演艺等产业发展，推进莲池中关村数字文物文化产业园、竞秀大激店世界音乐节、清苑正迪欢乐世界、高碑店中新健康城攀岩基地等项目建设。加强“保定老字号”保护和发展，支持市文化产业发展集团、直隶大剧院等文化龙头企业做大做强。合理布局养老机构与老年病医院，全面放开养老服务市场，打造面向京津的医疗保健、健康养老产业集群。

（四）强力推进城乡统筹发展，提高综合承载能力。瞄准京津冀世界级城市群大格局，加强规划、建设、管理，统筹生产生活生态，实现宜居宜业宜游。

做大中心城市。拉大城市框架，做大城市规模，增强辐射带动，打好城市建设攻坚战。加快对接融合。推进东三环南延、北延，乐凯大街、西三环南延，北三环、复兴路、七一路西延。推动恒祥大街北延、裕华路东延、境内107国道改线。谋划南三环、城市快速路及立交桥建设。推进公交都市试点建设，开通大王店至清苑、满城至高铁站快速无轨电车，启动市区地铁、轻轨建设项目的前期工作。打造四大板块。东湖和高铁板块，完成博物馆、东湖公园建设，规划馆、关汉卿大剧院投入使用，启动图书馆、档案馆建设。西部文体新城板块，完成西湖体育中心集中安置区建设，启动西湖公园、体育场馆建设，加快交通路网改扩建工程，建设一亩泉万亩森林生态隔离带。南部长城汽车科技城板块，编制专项规划，加快城中村拆迁改造和安置房建设，推进智慧新城核心区建设。北部低碳新城板块，完成城市控规修编和城市设计规划编制，启动区域交通路网、漕河景观带、生态隔离区和市民服务中心建设。加强历史文化名城建设，推进淮军公所、清河道署、直隶审判厅的保护利用。加快推进菊胡同、大世界等棚户区拆迁改造。

推进县城建设。坚持小县大县城战略，深化“县城建设三年攻坚”行动。涞源、阜平完成省级创园，涿州、高碑店、安国、定兴、涞水率先创建国家级园林县城。加强县城基础设施和公共服务设施建设，推进产城教一体化发展。制定重点特色产业目录，整合扶持生产要素，打造县域经济的四梁八柱。借鉴“京西百渡”经验，推进高碑店、定兴和白沟新城，涞水、易县和涞源，高阳、蠡县和博野，曲阳、阜平和唐县区域性组团发展，支持涞水与房山共建京冀新型城镇化协调发展示范区。抓好京冀交界地区和雄安新区周边的管控，对相关区域范围内的产业发展、城镇建设和生态环保进行有效管控，严防出现房地产“围城”问题。

培育特色城镇。择优培育一批重点中心镇和特色小镇，促进人口和产业聚集。抓好高阳庞口、定兴固城、涿州松林店等重点中心镇建设，抓好省定首批创建和培育的12个特色小镇[19]，全市重点打造20—30个特色小镇，每县创建1—2个。推进新型农村社区、产业园区、生态园区融合建设，以鲜活特色形成“名镇效应”，逐步把城市周边的小城镇发展成卫星城镇，远离城市的小城镇发展成综合型小城镇。

建设美丽乡村。制定完善县（市）域乡村建设规划、美丽乡村片区和村庄规划，加快推进保留村就地改造、中心村联村并建、特色村保护开发、撤并村整合搬迁。强化县级主体责任，构建“政府引领、农民参与、社会帮扶、市场运作”推进机制，年内完成省定379个重点村建设任务。推动建筑设计下乡，试聘乡村规划师，开展田园建筑示范，突出田园风貌和乡土特色。用足用好城乡建设用地政策，增减挂钩指标收益全部返还支持农业农村发展。实施农村电网改造工程，新增节水灌溉面积30万亩，改造农村公路2000公里。

创新城乡管理。落实《保定市城市市容和环境卫生条例》，突出抓好城市绿化、净化、亮化、秩序化。提升城市主要节点、干道绿化品质，小街小巷增绿添绿。推进市、县“洁净城市”创建，环境卫生整治重点向城中村、棚户区、小街巷、城乡结合部延伸，实现生活垃圾全链密闭化处理。开展农村“两改一清一拆”[20]行动，集中治理垃圾围村问题。实施城市亮化工程，打造明暗结合、动静相宜、简约典雅的城市亮化景观。建设一批智慧城市项目，年内实现城区（含县城）公共区域免费WiFi全覆盖。整合新建一批农贸市场，逐步取缔马路市场、外溢市场，抓好火车站、汽车站、高速口、校园、公园、大型商场等重点部位及周边地区的环境综合整治，让保定城市更干净、更整洁、更有序、更文明。

（五）强力推进大气污染防治，改善生态环境。生态环境没有替代品，用之不觉、失之难存。坚持生态优先、绿色发展，建设天蓝、地绿、水清的美好家园。

实施精准治霾。全面落实省委、省政府《关于强力推进大气污染综合治理的意见》，深入实施《保定市大气污染防治条例》和《大气污染防治九项治本措施》，推进大气污染治理十大工程[21]，全民动员开展“蓝天行动”。以能源替代为核心抓好禁煤控煤。继续实施煤改电改气工程，保北禁煤区完成1057个村、40余万户任务,除电厂、集中供热和原料用煤外，10月底前实现燃料煤归零；环主城区禁煤区年底前率先完成三环以外至高环以内234个重点村、约15万户改气改电任务。市县建成区集中供热及清洁能源供热率达到100%。全市范围10蒸吨燃煤锅炉和主城区35蒸吨燃煤锅炉实现清零。以硬化绿化净化为重点抓好抑尘降尘。主城区二环内消灭裸土，二环外、三环内道路全部硬化，严控工地扬尘。实施城市保洁网格化管理，落实降尘门前三包，城市垃圾及时快速清运，取缔砂石料摊点和废品收购站。以治污减排为抓手清理整治“小散乱污”企业。全部淘汰钢铁产能，加快淘汰铸造、水泥等落后产能，深度治理非金属矿山，强化挥发性有机物治理，提升餐饮油烟治理水平，取缔土小污染企业。推行国六标准车用柴油、汽油，持续抓好机动车污染治理。

加强生态修复。划定生态保护红线，落实生态保护空间。全面推行“河长制”，加强河流污染治理、河道管理和白洋淀流域综合整治。实施中小河流、水土保持项目建设及城区水系水生态综合整治。推进北拒马河综合治理，与北京房山区共建京西南生态示范核心区。加强农村面源污染治理，重点治理生活污水、生活垃圾、畜禽污染，抓好秸秆禁烧和综合利用，加强土壤污染检测和生态修复。推进“三网”“四带”“五园”[22]建设，实施三北防护林、太行山绿化和国家储备林项目，构建京津保生态过渡带。完成人工造林50万亩、封山育林及飞播造林20万亩，森林覆盖率提高1.45个百分点。

强化监管问责。实施“雷霆行动”，出重拳治理污染，用重典严格执法，构建最严责任体系，建立最严监控机制，落实最严执法监管，做到最严追责问责。实行数字化立体监测监管，扩大网格化监测区域，利用天眼工程、无人机、环境监测车实施立体监控。用足用好法定权力，利剑斩污，对破坏生态、污染环境的违法犯罪严厉打击、绝不手软。

（六）强力推进改革开放，释放发展活力。全面优化发展环境，在改革创新、开放合作中争取最大红利。

全面深化改革。深化行政体制改革，升级“两个代办”，以清单管理推动减政放权。创新开发区管理机制，深化人事、薪酬制度改革，提高管理建设水平。深化国资国企改革，实现国有企业信息披露、经营业绩考核和巡察全覆盖，加快混合所有制改革，推进国有企业“三供一业”分离[23]。加快财政事权和支出责任划分改革，深化绩效预算管理改革，推进预决算公开。做好“营改增”后续工作，实行综合与分类相结合的个人所得税改革。深化养老保险制度改革，完善个人账户制度，推行企业年金、职业年金、商业养老保险制度。推进农村集体产权制度改革试点，深化农村土地制度“三权分置”改革，落实集体所有权、稳定农户承包权、放活土地经营权。完成农村承包土地确权登记，加快农村宅基地和集体建设用地确权登记颁证。鼓励通过农村闲置宅基地整理、土地整治等增加耕地和建设用地，采取入股、联营等方式，重点用于乡村休闲旅游、养老等产业发展。实现县级农村产权流转交易市场全覆盖。深入推进社会领域、要素资源、价格机制、林权林场等各项改革。

全面扩大开放。制定出台招商引资新政策、新机制、新办法，紧盯先进制造业、战略性新兴产业、现代服务业等领域，瞄准国内外500强和行业领军企业，吸引更多优质项目落户保定。支持发展总部经济、会展经济、楼宇经济。转变招商观念，实施“互联网＋招商”、代理招商、定点招商、产业招商、以商招商、乡情招商，增强招商实效。健全招商引资目标责任制，层层分解任务、传导压力。支持开发区引入专业化园区运营商，把开发区建成对外开放的主战场和示范区。加大重点出口企业扶持力度，加强出口基地建设，支持本地企业走出去拓展发展空间。

全面优化营商环境。持续打好优化营商环境组合拳，着重解决程序繁、盖章多、部门不衔接等问题，建设全市统一的互联网＋政务服务平台。年内市县全部组建行政审批局，促进行政审批规范化、标准化、制度化。深化商事制度改革，扩大“多证合一、一照一码”[24]覆盖范围，降低准入成本，激发市场活力，年内各类市场主体达到60万户。构建信用监管平台，完善信息共享机制，推进市场主体守信联合激励、失信联合惩戒。加强知识产权保护，维护企业合法权益。推行“双随机一公开”[25]，整顿规范市场经济秩序，打造公平高效的市场环境。拓展清单管理范围，规范各类中介服务，对涉及群众办事的“奇葩证明”“循环证明”集中清理。完善服务企业恳谈会、双周重点项目调度会制度，激励企业创业创新创富，尊重保护企业家，使企业安心投资、宁静经营。弘扬亲商文化，持续叫响“来到保定、一切搞定”。

（七）强力推进精准扶贫脱贫，提高脱贫质量。落实“六个精准”[26]“五个一批”[27]要求，实施“绣花式”扶贫，打好脱贫攻坚硬仗，年内确保13万贫困人口稳定脱贫。

推进深度扶贫。发挥政府主导、群众主体、市场引导、社会帮扶、基层保障“五个作用”，对阜平、涞源等深度贫困县和深度贫困村，采取“五包一”“三包一”“一包一”[28]帮扶措施，在领导力量上、资金投入上、项目摆放上、政策扶持上、协同对接上、人才支持上重点倾斜，集中力量啃硬骨头，推进深度贫困地区脱贫攻坚。规范管理医保、养老、低保、五保、农业综合补贴和扶贫手册、政策宣传册“五证两册”，确保不落一户全部发放到位。强化社会兜底，做到应保尽保、应扶尽扶。推进新型社区、产业园区和生态园区“三区同建”，启动6.18万人易地扶贫搬迁工作，38个集中安置区建成入住。

突出产业扶贫。因人因户因村施策，发展培育特色种植养殖、家庭手工业、光伏、旅游等项目，实现贫困村、贫困户扶贫产业全覆盖。统筹技术、市场、加工环节，实施龙头带动，加强园区建设，拓展精深加工，协助产销对接，延长产业链条，强化服务保障，确保贫困群众在产业发展中获得长期稳定的收益。重点建设金融融资、电商、技术服务、资产收益和股份合作五大扶贫平台，增加贫困群众资产性收入，增强贫困地区自我发展能力。

健全脱贫机制。健全和完善政策保障、工作帮扶、干部激励和稳定脱贫保障四项机制，推进京保扶贫协作，加强与中直、省直、驻军等帮扶单位对接服务，鼓励经济强县、民营企业结对帮扶。扎实推进交通、水利、生态、教育、健康等扶贫行动，统筹脱贫攻坚、现代农业、山区综合开发、乡村旅游和美丽乡村“五位一体”建设，探索财政扶贫资金增长、涉农资金整合、土地扶贫政策、生态补偿脱贫等多种途径，壮大提高贫困村集体收入水平，扶持特殊贫困群众实现脱贫。强化脱贫考核机制，从严考核监督，严格贫困退出，防止数字脱贫、虚假脱贫。

（八）强力推进社会事业发展，改善民生福祉。始终把百姓冷暖放在心头，时刻把改善民生责任扛在肩上，切实把惠民利民实事落到实处，让人民群众有更多获得感、幸福感。

做好就业和社会保障工作。实施高校毕业生就业创业促进计划，做好军队转业干部和退役士兵接收安置工作。搞好农民工职业技能培训，支持农民工返乡创业。年内新增就业9万人、再就业2.69万人。严厉打击恶意欠薪行为，有效保障劳动者合法权益。继续提高最低生活保障标准，城市提高到每年6000元，农村提高到每年3360元。加大医疗救助和临时救助力度，着力解决因病因灾生活困难问题。稳步增加企业职工收入，提高养老保险待遇，确保养老金按时足额发放。实施全民参保计划，基本医疗保险参保率稳定在95%以上。加快棚户区住房改造，全年完成17432套（户）。扩大限价房、廉租房、公租房等保障性住房供给，建立购租并重住房体系。坚持“房子是用来住的、不是用来炒的”定位，去除房产投机属性，回归房屋居住功能，采取限购限贷措施，加大住宅用地供应，规范房产市场秩序，坚决把房价控制在合理区间。

发展各项社会事业。继续推进县城和市区义务教育的两个“三年行动计划”[29]，县城新改扩建学校30所，市区启动建设4所。启动5个教育聚集区建设，新改扩建5所普通高中、150所幼儿园，改造提升农村义务教育学校55所。提高教育质量，探索集团化办学，推进义务教育均衡发展。推进校园新风净化工程建设，开展小学生课后托管服务试点工作。推进公立医院综合改革，加快北京安贞医院保定分院、北京中医院安国分院等项目建设，打造中医药强市。扎实推进书香保定建设，构建现代公共文化服务体系。加强体育设施建设，推进全民健身，办好赛事活动。加强国防教育和国防后备力量建设，继续推进双拥共建，优化军民融合发展环境。积极维护民族团结，全力促进宗教和谐。继续做好广播电视、新闻出版、外事侨务、气象地震、防灾减灾、邮政通信、史志档案和老龄、妇女儿童、残疾人等工作。

确保社会安全稳定。加强社会治安综合治理，持续开展严打整治行动，保持对违法犯罪的高压态势。完善预警和应急预案，增强重大突发敏感事件处置能力。强化安全生产监管，确保食品药品安全。积极开展卫生城市、洁净城市、人文城市和文明城市“四城同创”，成功创建省级文明城市，切实提升城市美誉度。

各位代表，去年我市确定的55件惠民实事基本完成。为了多谋民生之利、多解民生之忧，在多渠道多层次广泛征求群众意见的基础上，市委、市政府确定今年要办好10个方面58项惠民实事。我们一定想方设法筹措资金，千方百计克服困难，努力把实事办实、好事办好，向全市人民交上一份满意的答卷。

全面加强自身建设

转变作风真抓实干建设为民务实清廉的法治政府

实干成就梦想，奋斗再铸辉煌。新一届政府将紧紧围绕一个“实”字，死死咬住一个“干”字，坚持说实话、干实事，用实招、使实劲，真正把人民代表议定的事项落到实处，着力打造为百姓造福、让人民满意的法治政府。

讲政治，增强发展凝聚力。牢固树立“四个意识”，自觉与以习近平同志为核心的党中央保持高度一致，做到思想上拥戴核心、政治上信赖核心、组织上忠诚核心、行动上捍卫核心，做到对党忠心、对群众真心、对事业专心，精心、精细、精致、极致做好各项工作。主动加强学习，深入调查研究，克服“本领恐慌”，摆脱“路径依赖”，提高领导发展能力。增强责任感、使命感，弘扬主旋律，凝聚正能量，不折不扣把上级决策部署落到实处。

重法治，增强政府公信力。忠实履行宪法和法律赋予的职责，自觉运用法治思维和法律方式推动工作。坚持科学民主决策，自觉接受人大法律监督、政协民主监督和社会公众监督。认真办理人大代表建议和政协提案，坚持重大事项向人大报告制度和向政协通报制度。坚持以权力瘦身为廉政强身,紧紧扎住制度围栏,坚决打掉寻租空间,努力铲除腐败土壤。坚持“法无授权不可为、法定职责必须为”,依法履责、规范行政、公正执法、文明用权。落实行政执法公示、执法全过程记录、重大执法决定法制审核制度，维护社会公平正义。

转作风，增强工作执行力。深化机关作风整顿，把“马上就办、办就办好”作为基本准则，凡是涉及民生、百姓关心的事情，都雷厉风行，盯住干，马上办。改进文风会风，把精力用在抓落实上、把功夫下在见实效上。倡导创新精神，焕发最佳精神状态，以燕子垒窝的恒劲、蚂蚁啃骨的韧劲做好各项工作。完善责任机制和督查机制，“脸难看、话难听”不行，“话好听、事不办”也不行，对为官不为、工作不力的严肃问责，对懒政怠政、失职渎职的严厉查处。

树形象，增强社会感召力。坚持从严治政，始终把纪律和规矩挺在前面，深入推进政府系统党风廉政建设，规范政府的行政权力和行为，营造风清气正的政治生态，以党风政风带民风，促进社会风气的进一步好转。严格执行《准则》和《条例》，严格落实“两个责任”和“一岗双责”，强化行政监察和审计监督，认真纠正部门和行业的不正之风，严肃查处各类违纪违法案件，毫不手软地依法惩处腐败分子。健全激励机制和容错纠错机制，给干事者鼓劲，为担当者撑腰，上下同欲、众志成城，合力顶起保定跨越、崛起和复兴的这片蓝天。

各位代表！潮平岸阔催人进，风正帆悬正当时。让我们紧密团结在以习近平同志为核心的党中央周围，在省委、省政府和市委的正确领导下，发扬“赶考”和“长征”精神，携手河北雄安新区，撸起袖子加油干，扑下身子真作为，以良好的精神状态和出色的工作业绩，迎接党的十九大胜利召开，为加快建设创新驱动经济强市、推动京津保地区率先联动发展、全面建成小康社会、加快实现保定复兴而努力奋斗！
